# Acupuncture for adult lung cancer of patient-reported outcomes: A systematic review and meta-analysis

**DOI:** 10.3389/fonc.2022.921151

**Published:** 2022-09-02

**Authors:** Ziqi Xi, Xuqiang Wei, Zi Ye, Ke Wang, Jia Zhou

**Affiliations:** Acupuncture Anesthesia Clinical Research Institute, Yueyang Hospital of Integrated Traditional Chinese and Western Medicine, Shanghai University of Traditional Chinese Medicine, Shanghai, China

**Keywords:** acupuncture, lung cancer, PROs = patient-reported outcomes, systematic review, meta-analysis

## Abstract

**Purpose:**

This systematic review and meta-analysis aims to assess the effects of acupuncture on patient-reported outcomes (PROs) in adults with lung cancer.

**Methods:**

Electronic databases including PubMed, Embase, Cochrane Library, Web of Science, China National Knowledge Infrastructure (CNKI), China Science and Technology Journal Database (CQVIP), Wanfang Data, SinoMed, and gray literatures were retrieved from inception to 1 July 2022 for randomized controlled trials (RCTs). Acupuncture was defined as an experimental intervention, and the patients of the control groups included either treatment including conventional therapy (usual care, sham/placebo acupuncture, pharmacotherapy including Western medicine and Chinese traditional medicine). PROs for this study were measured by seven scales of primary outcomes including the Karnofsky Performance Status (KPS), European Organization for Research and Treatment of Cancer Quality of Life Questionnaire, Functional Assessment of Cancer Therapy-Lung, Functional Assessment of Cancer Therapy Lung Cancer Subscale, Leicester Cough Questionnaire (LCQ score), the Medical Outcomes Study (MOS) item short form health survey (SF-36), and the St George’s Respiratory Questionnaire, and 12 scales of secondary outcomes. Cochrane Collaboration’s tool was used to assess the risks of bias. Data were combined and analyzed with RevMan 5.4 and Stata/SE 16.0.

**Results:**

We retrieved 3,002 lung cancer patients from 33 trials. KPS included with 1,000 patients showed that acupuncture could significantly improve the quality of life (QOL) compared with the control group regardless of different tumor–node–metastasis stages or the different stages of disease. The study showed that acupuncture significantly improved lung cancer–related symptoms in the QOL, pain, nausea and vomiting, insomnia, anxiety and depression, fatigue, and constipation compared with the control group. Eight RCTs reported the occurrence of adverse events, whereas four reported none and four RCTs reported that the events in the observation group were significantly less than those in the control group.

**Conclusion:**

Acupuncture proved to be a promising intervention, both postoperatively and after chemotherapy, and should be recommended as a beneficial alternative strategy to promote PROs in lung cancer patients at all stages of application. Considering the low quality, we suggest more rigorous clinical trials of acupuncture for lung cancer in the future and more emphasis on the effect of acupuncture in patients with lung cancer on their PROs, mainly in the aspect of the QOL.

**Systematic review registration:**

https://www.crd.york.ac.uk/prospero/display_record.php?, identifier [CRD42021274122].

## Introduction

Lung cancer remains the most common cancer and the leading cause of cancer deaths (1). The overall 5-year survival rate for lung cancer diagnosed from 2010 to 2014 was in the range 10%–20% in most countries around the world, still being dismal (2). According to the latest global statistical analysis of International Agency for Research on Cancer, approximately 2.2 million new cases were diagnosed worldwide in 2020, with a mortality rate of 18% in the same year (3). Furthermore, the cost of drugs imposes a heavy social and economic burden on individuals, families, communities, and countries, thus posing substantial challenges (4). Studies have been conducted on patients after lung cancer surgery, commonly showing a significant decline in the quality of life (QOL) scores (5, 6).

Patient-reported outcomes (PROs) are the measurements of any aspect of a patient’s health obtained by a self-report, which means that there is no need for physician or any others to interpret the patient’s reactions (7). PROs are becoming increasingly important in the evaluation of cancer treatment modalities (8). PROs provide valuable insight into the patient experience and allow the measurement of preoperative and postoperative QOL (9). QOL is a critical outcome measure in lung cancer surgery and is of great significance, especially in treating patients with early-stage lung cancer (10). It has been reported that PRO-based active symptom monitoring intervention is feasible and demonstrates encouraging preliminary efficacy for reducing symptoms and the readmission risk (11), and more to the point, resulting in superior QOL (12).

Recent advances in clinical research show that acupuncture, as an effective, safe, and cost-effective treatment for cancer and cancer-related symptoms, may provide clinical benefits for oncology patients in symptom control and supportive care (13, 14). Acupuncture also alleviates side effects induced by chemotherapy or radiotherapy such as nausea and vomiting (15), cancer-related pain (16), fatigue (17), insomnia (18), and the QOL. Oncology acupuncture has become a new research field with great prospects (19). It is anticipated that as a growing number of evidence continues to emerge, oncology acupuncture will eventually be integrated into standard oncology practice (20).

Despite growing attention to acupuncture as an alternative medicine for lung cancer treatment, the evidence of its impact on the PROs of lung cancer patients is scanty (21). Moreover, there are no systematic reviews of acupuncture improving PROs in lung cancer patients. To fill this gap, we undertake systematic retrieval and analysis to summarize the existing evidence of acupuncture therapy in improving PROs among the lung cancer patients. Our study will provide more reliable evidence from the perspective of PROs and the implementation details of acupuncture therapies in the clinical practice of lung cancer, as well as contribute to optimizing a clinical acupuncture regimen and trial design in the future.

## Methods

This study is performed according to the Preferred Reporting Items for Systematic Review and Meta-Analyses (PRISMA) guidelines (22). The protocol of this study has been registered in International Prospective Register of Systematic Reviews (PROSPERO), and the registration number is CRD42021274122.

### Search strategy

Electronic databases including PubMed, Embase, Cochrane Library, Web of science, CNKI, CQVIP, Wanfang Data, SinoMed, and gray literatures including ClinicalTrials.gov Database (www.clinicaltrials.gov), Chinese Clinical Trial Register (www.chictr.org.cn), and conference literatures were retrieved from inception to 1 July 2022. The language is limited to Chinese and English. In addition, the reference lists of eligible articles were also checked to identify additional studies. The searches were performed using the following mesh terms plus keywords, such as “acupuncture”, “lung cancer”, “PROs”, and “Randomized Controlled Trial”, including their synonyms. **Supplementary Tables 1**, **2** in the **Supplementary Materials** show the complete search strategy for English and Chinese databases above.

### Inclusion criteria

The eligible criteria included:

- Adult patients (age ≥ 18 years) who were diagnosed with lung cancer through pathology with any tumor stage with no gender restrictions
- Randomized controlled trials (RCTs) on acupuncture treatment among lung cancer patients with the outcomes of PROs
- Acupuncture is a method to treat diseases by stimulating meridians and acupoints. It includes manual acupuncture, electroacupuncture (EA), moxibustion, transcutaneous electrical acupoint stimulation (TEAS), auriculotherapy, acupoint application, acupoint injection, fire needle, plum-blossom needle, and acupressure. Acupuncture used alone or in combination was defined as an experimental intervention.
- The comparison groups included either treatment as follows: usual care, sham/placebo acupuncture, and pharmacotherapy including Western medicine (WM) and Chinese Traditional medicine (TCM).

### Exclusion criteria

The eligible criteria included:

- Combined with other cancers
- Quasi-randomized control trial, cohort studies, case–control studies, and articles that have not been peer-reviewed
- The same acupuncture therapy was conducted in both groups.

It deserves to be mentioned that the acupuncture group has no restriction on the needle size, acupoint selection, stimulation frequency, retention time, and treatment course.

### Outcome measures

We divided the different PRO outcome indicators into two categories: QOL and patient-perceived symptoms. The primary outcome measures were QOL scales commonly used in the efficacy evaluation of lung cancer patients, such as the Karnofsky Performance Status (KPS) (23), European Organization for Research and Treatment of Cancer Quality of Life Questionnaire (EORTC QLQ-C30) (24), Functional Assessment of Cancer Therapy-Lung (FACT-L) (25), Functional Assessment of Cancer Therapy Lung Cancer Subscale (FACT-LCS), Leicester Cough Questionnaire (LCQ score) (26), the MOS item short form health survey (SF-36) (27), and the St George’s Respiratory Questionnaire (SGRQ) (28).

Secondary outcomes were patient-perceived symptoms, including pain, nausea and vomiting, insomnia, fatigue, and constipation, as well as adverse events to be recorded. Pain intensity was measured by four measurement tools including the numerical rating scale (NRS) score (29), Visual Analog Scale (VAS) pain scales (30), pain score in EORTC QLQ-C30, and Brief Pain Inventory-Chinese Version (BPI-C) (31). Nausea and vomiting were measured by three measurement tools including the MASCC (Multinational Association of Supportive Care in Cancer) Antiemesis Tool (MAT) (32), Index of Nausea and Vomiting and Retching (INVR) (33), and nausea and vomiting score in EORTC QLQ-C30. Insomnia was measured by three measurement tools including the Pittsburgh Sleep Quality Index (PSQI) (34), Athens Insomnia Scale (AIS) (35), and sleep score in EORTC QLQ-C30. Fatigue was measured by the following four measurement tools: Revised Piper Fatigue Scale (PFS-R) (36), Brief Fatigue Inventory-Chinese Version (BFI-C) (37), and fatigue score in EORTC QLQ-C30. The secondary outcome anxiety and depression was measured by two measurement tools including the Self-Rating Anxiety Scale (SAS) (38) and Self-Rating Depression Scale (SDS) (39). Constipation was measured by following one measurement tool constipation score in EORTC QLQ-C30.

### Study selection and data extraction

Two researchers (X.Q. W and Z.Q. X) independently extracted and managed data by Excel software (16.59, Microsoft excel for Mac). Any disagreement was resolved by discussion until a consensus was reached or by consulting a third researcher (K W). The data extraction elements included the authors, year, sex, age, stage, randomization, intervention details, main acupoint, course of treatment, results, follow-up, and outcomes. For RCTs with multiple time points to evaluate outcomes, the data at the end of treatment were extracted. The selection process was presented in a PRISMA flow diagram.

### Risk of bias assessment

Methodological quality and reporting biases were evaluated by two reviewers independently. Cochrane Collaboration’ s tool was used to assess the risks of bias (40). We assessed from the following seven dimensions: random sequence generation, allocation concealment, the blinding of participants and patients, the blinding of outcome evaluators, incomplete outcome data, and selective reporting. Divergence would be conquered by the adjudication of the corresponding author.

### Statistical analysis

Data were combined and analyzed with RevMan 5.4.1 (The Cochrane Collaboration) and Stata/SE 16.0. Dichotomous data were reported as the relative ratio (RR), whereas continuous data were reported as the mean difference (MD) or standardized mean difference (SMD), with 95% confidence interval (CI). The MD was used for PROs with the same measures; otherwise, the SMD was chosen. The fixed-effect model was employed when the study of heterogeneity (I^2^) was <50%; otherwise, a random-effect model was used. Sensitivity analysis was performed by excluding each RCT out sequentially to test the robustness of the result. Subgroup analysis was applied to explore the source of heterogeneity. Meta regression analysis was used to clarify the sources and value of heterogeneity and to further explain the influence of variables on the combined effect. A random-effect model was used for meta regression analysis. The funnel plot was conducted to detect publication bias.

## Results

### Search results

**Figure 1:**
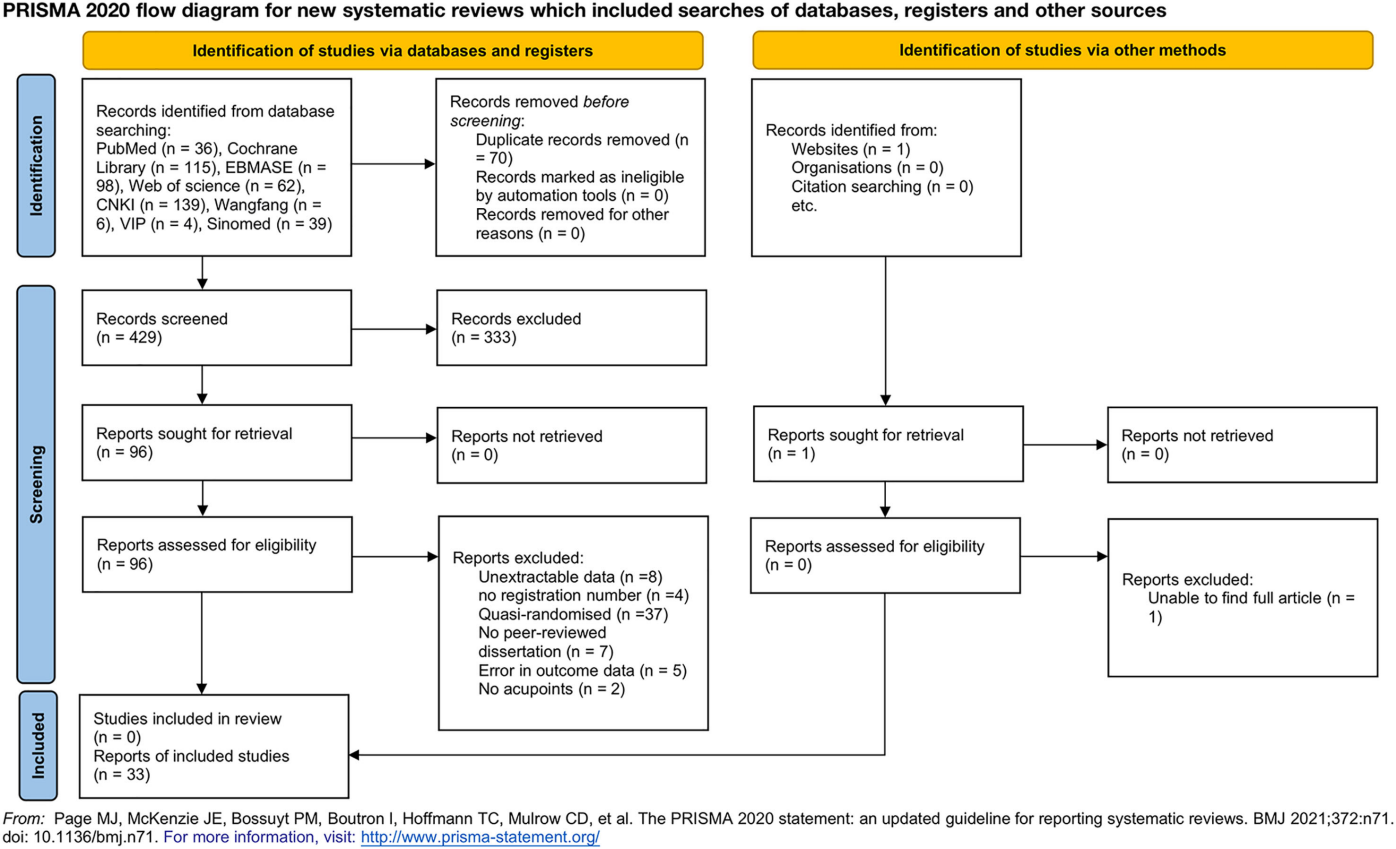
Preferred Reporting Items for Systematic Review and Meta-Analyses (PRISMA) 2020 flow diagram for new systematic reviews that included the searches of databases, registers, and other sources. From: Page MJ, McKenzie JE, Bossuyt PM, Boutron I, Hoffmann TC, Mulrow CD, et al. The PRISMA 2020 statement: An updated guideline for reporting systematic reviews. BMJ 2021;372:n71.doi:10.1136/bmj.n71. For more information, visit http://www.prisma-statement.org/.

A total of 499 trials were retrieved in the literature search. After a preliminary screening of the titles and abstracts of the articles, we used EndNoteX9 (X9.3.3, Thomson Reuters (Scientific) LLC Philadelphia, PA, USA) and manual checking to remove duplicate and non-standard studies and identified 96 studies from the database. We evaluated the full text of the 96 studies, and only 33 of them met our inclusion criteria. **Figure 1** shows the process of the literature search and the screening process used in this study (22).

### Descriptions of the included trials

Of all included 33 RCTs, 27 were published in Chinese and 6 were published in English. Seven trials used auricular acupoint (41–47), seven trials used acupoint application (45, 46, 48–52), six trials used manual acupuncture (41, 46, 53–56), four trials used moxibustion (43, 48, 57, 58), three trials used electroacupuncture (EA) (51, 59, 60), three trials used transcutaneous electrical acupoint stimulation (TEAS) (61–63), two trials used acupoint injection (49, 64), two trials used a fire needle (65, 66), two trials used acupressure (67, 68), and only one trial for each used plum-blossom needle tapping (69), low-frequency pulse (49), catgut-embedding therapy (70), thunder-fire moxibustion (71), thermal moxibustion (72), and Mongolian medicine warm acupuncture (73). All RCTs provided the details of the treatment acupoints. The auricular acupoint sessions ranged from 3 to 5 min. Plum-blossom needle tapping sessions ranged from 5 to 10 min. Acupressure sessions ranged from 10 to 18 min. Thunder⁃fire moxibustion sessions ranged from 20 to 30 min. Low-frequency pulse sessions ranged from 25 min. Moxibustion, EA, TEAS, manual acupuncture, and Mongolian medicine warm acupuncture sessions ranged from 30 min, and acupoint application sessions ranged from 4 to 6 h. Fire needle sessions were done three times at each point.

Twenty trials used Western medicine or traditional Chinese medicine (TCM) as a control intervention, 9 trials used usual care as a control intervention, and only 4 trials used sham or placebo acupuncture.

Nineteen trials covered the primary outcomes, 8 of which also included secondary outcomes. The remaining 14 trials only with secondary outcomes. The characteristics of the included studies and acupuncture details of included studies are shown in **Supplementary Tables 3** and **4** in the Supplementary Materials.

### Risk of bias in individual trials

**Figure 2:**
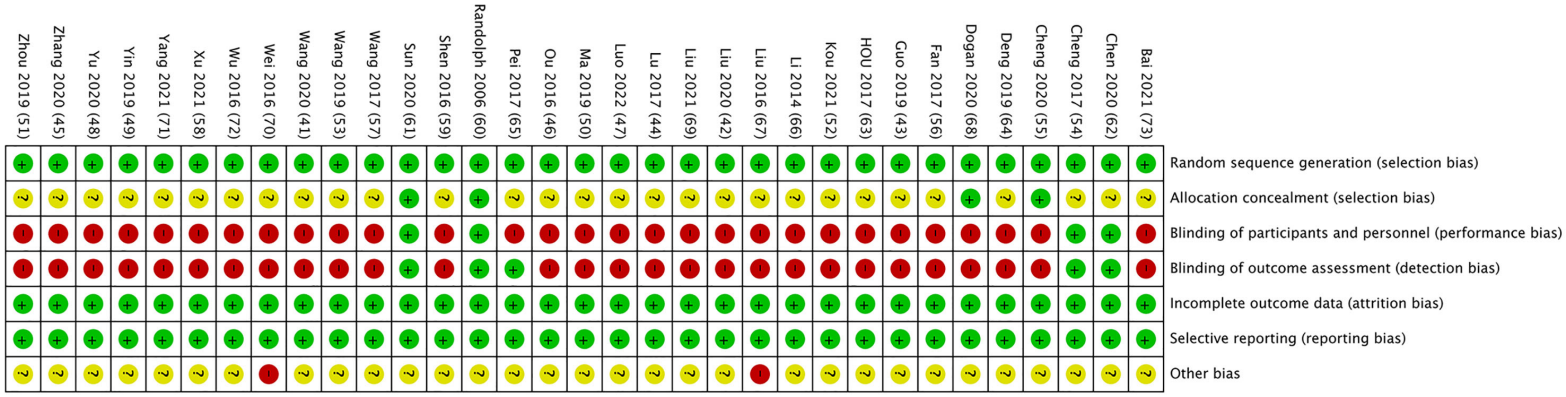
Risk of bias assessment by individual trials.

All of the RCTs reported the generation of random sequences. Twenty-three trials used the random number table method, five trials used random number produced by computer, two trials used lottery, two trials used random number produced by SPSS (20.0 and 22.0) and the remaining one trial used regional random grouping method. The major sources of risk of bias correlated with allocation concealment, blinding of participants and personnel, and blinding of outcome assessment. twenty-nine trials were judged to have a high risk of bias with respect to the blinding of participants given that it was not possible to blind the acupuncturists and most patients in a study of acupuncture intervention. Other bias in two trials (67, 70) were identified as high risk because these two trials did not report baseline data for age comparison of patient in both control group and observation group. We judge that only two trials (60, 61) had a relatively low risk of bias. The individual risk of bias for each trial is presented in **Figure 2**.

### Outcome measures

#### Quality of life

Seventeen RCTs (41, 42, 45–50, 53, 54, 57, 58, 61, 64–66, 69) reported QOL scales, including KPS (42, 45–50, 53, 61, 64–66, 69), EORTC QLQ-C30 (41, 57), FACT-L (61, 65), FACT-LCS (54), SF-36 (58), LCQ (50), and SGRQ (68). Among these 17 trials, 7 trials used ST36; 4 trials used BL13, LI4, and TF4; 3 trials used PC6 and AH6a; and 2 trials used AT4. Higher scores were considered better in all PROs except SGRQ in which a higher score indicates a poorer QOL. In terms of the QOL measured by KPS, 11 of which used continuous variables and two original studies referred to the criteria of KPS and transformed it into a dichotomous variable. We combined these trials in two different ways: different tumor–node–metastasis (TNM) stage or different stage of disease. A study (74) has shown that KPS appeared to be a more reliable predictor of survival than the results of the QOL questionnaire. This again suggests that KPS is more informative when used to evaluate older and more impaired patients. It was found that the QOL of patients who received acupuncture-related treatment improved significantly compared with those who only received Western medicine treatment and usual care using continuous variables (MD 6.75, 95%CI, 5.82 to 7.68, P<0.00001, I^2^ = 0%) Two trials (46, 64) concluded that patients experienced a higher effect on QOL in the acupuncture-related group compared with those in the WM/TCM group using dichotomous variables (RR 1.24, 95%CI, 1.09 to 1.41, P=0.001 I^2 =^ 0%). The pooled analysis results of the changes in the mean scores of each scale are listed in **Tables 1** and **2**.

**Table 1:** The QOL of acupuncture with continuous variables versus comparators for lung cancer treatment–related symptoms.

Outcome	Participants	End of treatment				Meaning of higher scores
		IV, Fixed, 95% CI	P-value	Heterogeneity		
**KPS** (42, 45, 47–50, 53, 61, 65, 66, 69)						
**1. Different TNM stage**						
1.1 Early and middle stage (61)	120	MD 5.96 [1.79, 10.12]	P=0.005		/	Better
1.2 Late stage (47, 48)	170	MD 7.00 [5.65, 8.35]	P<0.00001		I^2^ = 0%	Better
1.3 Middle and late stage (42, 45, 50, 65, 66)	434	MD 6.75 [5.12, 8.37]	P<0.00001		I^2^ = 31%	Better
1.4 Not mentioned (49, 53, 69)	276	MD 6.28 [3.55, 9.01]	P<0.00001		I^2^ = 0%	Better
**2. Different stage of disease**						
2.1 Postsurgery (61, 69)	192	MD 6.12 [3.30, 8.93]	P<0.0001		I^2^ = 0%	Better
2.2 Undergoing chemotherapy (49, 53, 65, 66)	324	MD 7.09 [4.53, 11.25]	P<0.00001		I^2^ = 18%	Better
2.3 Not mentioned (42, 45, 48, 50)	396	MD 6.84 [5.67, 8.01]	P<0.00001		I^2^ = 0%	Better
**EORTC QLQ-C30** (41, 57)						
**1. Different TNM stage**						
1.1 T1–4 stage (41)	118	MD 13.00 [8.91, 17.09]	P<0.00001		/	Better
**2. Different stage of disease**						
2.1 Undergoing surgery (57)	96	MD 6.47 [-1.54, 14.48]	P<0.05		/	Better
**FACT-L** (61, 65)						
**1. Different stage of disease**						
1.1 Postsurgery (61)	120	MD 3.64 [0.32, 6.96]	P=0.03		/	Better
1.2 Undergoing chemotherapy (65)	60	MD 8.76 [2.05, 15.47]	P=0.01		/	Better
**FACT-LCS** (54)	28	MD 5.80 [4.63, 6.97]	P<0.00001		/	Better
**SF-36** (58)	100	MD 10.36 [6.17, 14.55]	P<0.00001		/	Better
**LCQ** (50)	120	MD 20.21 [15.61, 24.81]	P<0.00001		/	Better
**SGRQ** (68)	60	MD -31.69 [-36.58, -26.80]	P<0.00001		/	Worse

QOL, quality of life; TNM, tumor–node–metastasis; KPS, Karnofsky Performance Status; QLQ-C30, European Organization for Research and Treatment of Cancer Quality of Life Questionnaire; FACT-LCS, the Functional Assessment of Cancer Therapy Lung Cancer Subscale; SF-36, the MOS item short form health survey; LCQ, Leicester Cough Questionnaire; FACT-L, Functional Assessment of Cancer Therapy–Lung; SGRQ, St George’s Respiratory Questionnaire.

**Table 2:** The QOL of acupuncture with dichotomous variables versus comparators for lung cancer treatment–related symptoms.

Outcome	Participants	End of treatment			Meaning of higher scores
		M-H, Random, 95% CI	P-value	Heterogeneity	
**KPS**					
**Different TNM stage**					
Late stage (46)	142	RR 1.19 [1.00, 1.41]	P=0.009	/	Better
Middle and late stage (64)	80	RR 1.30 [1.07, 1.59]	P<0.05	/	Better

QOL, quality of life; KPS, Karnofsky Performance Status; TNM, tumor–node–metastasis.

#### Nausea and vomiting

Four RCTs (41, 51, 55, 57) reported on nausea and vomiting by patients and used three NA measures including the NV score in EORTC QLQ-C30 (41, 57), MAT (51) and INVR (55). Among these four trials, two trials used LI4 and ST36, one trial used TF4, AH6a, PC6, and AT4. Two (51, 55) of the trials were for nausea and vomiting after chemotherapy; one (57) was postoperative, and one (41) was not specified. Higher scores were considered worse in all these PROs. For NV measured by the NV score in EORTC QLQ-C30, Wang X et al. (41) and Wang LQ et al. (57) compared the NV scores of patients in the observation group after treatment with those in the control group; scores in the observation group were significantly lower than those in the control group for both postoperative and routine patients (MD -14.73, 95%CI, -23.88 to -5.59, P=0.002, I^2^ = 81%). In terms of INVR scores (55), there was no statistical difference between the POG and the control group on the first day of chemotherapy, but the prechemotherapy acupuncture group (PRG) differed significantly from the postchemotherapy acupuncture group (POG) and control group (P<0.05). On the second-to-seventh day of chemotherapy, the difference between the three groups was statistically significant (MD -1.18, 95%CI, -1.89 to -0.47, P=0.001).

One RCT (51) measured by MAT using dichotomous variables showed that the severity of acute vomiting in the observation group was significantly lower than that in the control group at the end of treatment (RR 0.59, 95%CI, 0.41 to 0.86, P=0.006) (**Table 5**).

#### Sleep disturbances

**Figure 4:**
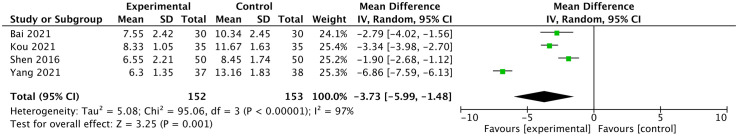
Forest plot of the sleep disturbances measured by Pittsburgh Sleep Quality Index in lung cancer patients treated with acupuncture and control.

Seven RCTs (41, 43, 44, 52, 59, 71, 73) reported on sleep disturbances by patients and used three sleep disturbance measures including the SL score in EORTC QLQ-C30 (41), PSQI (43, 52, 59, 71, 73), and AIS (44). Among these seven trials, three trials used TF4, AT4, and ST36; two trials used AH6a and LI4; and one trial used BL13. Three (43, 71, 73) of the trials were for sleep disturbances after chemotherapy, three (41, 52, 59) were cancer-related sleep disturbances, and one (44) was for radiotherapy. Higher scores were considered worse in this PRO. Five trials used PSQI to measure patients’ sleep quality, one of which (43) used four arms, so the results are not easy to be combined and will be analyzed separately. Guo et al. showed that there were statistically significant differences in the PSQI factor scores of the four groups, suggesting that the auricular acupoint combined with the moxibustion treatment group had the most obvious effect. The remaining four trials showed that the score of PSQI in the observation group was significantly lower than those in the control group and the total improvement rate of sleep quality was also superior to the patients in the control group for both chemotherapy-induced and cancer-related sleep disturbances (MD -3.73, 95%CI, -5.99 to -1.48, P=0.001, I^2^ = 97%) (**Figure 4**). For the SL score in EORTC QLQ-C30 (41), a comparison between groups after treatment showed that the SL scores in the observation group after acupuncture observation were lower than those in the control group (MD -14.16, 95%CI, -20.91 to -7.41, P<0.001). One RCT (44) using AIS showed that AIS scores in the observation group and the control group were not statistically significant (P>0.05) (MD -0.17, 95%CI, -1.93 to 1.59, P=0.85) (**Table 5**).

#### Fatigue

**Figure 5:**
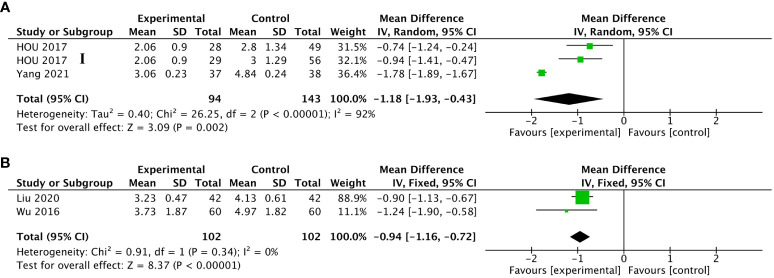
Forest plot of the fatigue in lung cancer patients treated with acupuncture and control. **(A)** The changes measured by the Revised Piper Fatigue Scale (PFS-R) in lung cancer patients undergoing chemotherapy treated with acupuncture-related intervention versus control from the baseline to the end of treatment; **(B)** the changes measured by PFS-R in lung cancer patients with cancer-related fatigue treated with acupuncture-related intervention versus control from the baseline to the end of treatment. IV, inverse variance; CI, confidence interval. The Roman numerals “I”, followed by the study ID, represented the comparison of acupuncture versus no intervention in the study that had three arms.

Eight RCTs (41, 42, 54, 57, 63, 67, 71, 72) reported on fatigue by patients and used four fatigue measures including the FA score in EORTC QLQ-C30 (41, 57), PFS-R (42, 63, 71, 72), and BFI-C (54, 67). Among these eight trials, five trials used ST36; three trials used LI4; two trials used AT4, BL13, TF4 and AH6a; and one trial used PC6. Three (63, 67, 71) of the trials were for fatigue after chemotherapy and five (41, 42, 54, 57, 72) were cancer-related fatigue. Higher scores were considered worse in all these PROs. Four RCTs used PFS-R to measure fatigue, including two (63, 71) trials of chemotherapy-induced fatigue and two (42, 72) trials of cancer-related fatigue. After weeks of intervention, the PFS score of the two groups was significantly lower than those before the intervention, and the decrease in the observation group was more significant than that in the control group for both chemotherapy-induced and cancer-related fatigue (MD -1.18, 95%CI, -1.93 to -0.43, P<0.00001, I^2^ = 92%) (MD -0.94, 95%CI, -1.16 to -0.72, P = 0.34, I^2^ = 0%) (**Figure 5**).

For FA measured by the FA score in EORTC QLQ-C30, Wang X et al. (41) and Wang LQ et al. (57) compared the FA scores of patients in the observation group after treatment with those in the control group; scores in the observation group were significantly lower than those in the control group (MD -12.81, 95%CI, -24.50 to -1.12, P=0.01, I^2^ = 84%). There were two trials measured by BFI-C using continuous variables and dichotomous variables, respectively. Cheng et al. (54) showed that patients who received active acupuncture had significantly lower BFI-C scores compared to those who received placebo (MD -1.40, 95%CI, -1.62 to -1.18, P<0.00001). Liu (67) showed that the degree of fatigue in the observation group was significantly lower than that of the control group (RR 0.84, 95%CI, 0.73 to 0.97, P=0.02) (**Table 5**).

#### Anxiety and depression

**Figure 6:**
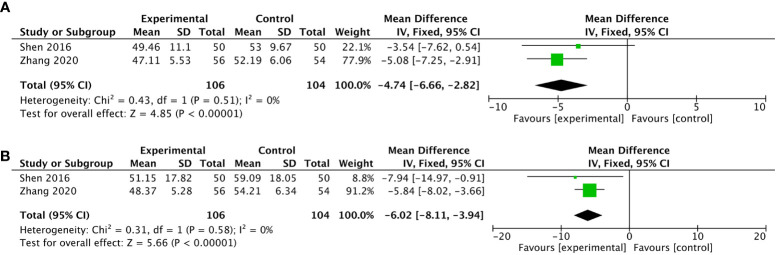
Forest plot of the fatigue in lung cancer patients treated with acupuncture and control. **(A)** the changes measured by the Self-Rating Anxiety Scale in lung cancer patients treated with acupuncture-related intervention versus control from the baseline to the end of treatment; **(B)** the changes measured by Self-Rating Depression Scale in lung cancer patients treated with acupuncture-related intervention versus control from the baseline to the end of treatment. IV, inverse variance; CI, confidence interval.

Two RCTs (45, 59) reported on anxiety and depression by patients and used two AD measures including SAS (45, 59) and SDS (45, 59). Among these two trials, BL13, TF4, AH6a, LI4, and ST36 were all used only once. Higher scores were considered worse in all these PROs. Shen et al. and Zhang et al. showed that compared with the control group, both SAS scores in the observation group were lower than those in the control group after treatment (MD -4.74, 95%CI, -6.66 to -2.82, P=0.51, I^2^ = 0%) and so were SDS scores (MD -6.02, 95%CI, -8.11 to -3.94, P=0.58, I^2^ = 0%) (**Figure 6**).

#### Constipation

Only one RCT (41) reported on anxiety and depression by patients and used the CO score in EORTC QLQ-C30. This trial used acupoints including LI4, LR3, AT4, TF4, and AH6a. Higher scores were considered worse in all these PROs. Wang et al. showed that the CO score of patients in the study group after treatment was lower than that in the control group (**Table 5**).

#### Side effect

Eight trials (42, 44, 46, 49, 50, 68, 70, 71) reported on side effects including dizziness, encephalalgia, fatigue, somnolence, gastrointestinal reaction, erythra, or respiratory depression. Three trials (46, 50, 71) reported no serious side effects in both groups. One trial (68) concluded that there were no serious side effects, but no data were available. Four trials reported that side effects in the observation group were lower than those in the control group and two (44, 70) of which had statistical significance (P<0.05), while the other two (42, 44) had no statistical significance (P>0.05). Since one patient could be associated with multiple side effects, and the author did not report in detail, the data were not convenient for statistics.

### Subgroup analysis

When we combined two secondary outcomes of pain, VAS and NRS, in the trials of cancer pain, the heterogeneity was up to 90%. Then, we compared the effects between subgroups according to the following methods: acupuncture technique, acupoint combination, frequency of treatment session, duration time, and TNM stage. The results are shown in **Table 3**.

**Table 3:** Subgroup analysis of the combination of NRS and VAS in patients with cancer pain.

Outcome	Subgroup	Participants	End of treatment		
			IV, Random, 95% CI	P value	Heterogeneity (I^2^)
**Pain**	**Acupuncture technique**				
	Manual acupuncture (56)	69	MD -0.61 [-1.12, -0.10]	P=0.02	**/**
	TEAS (59)	100	MD -2.12 [-2.55, -1.69]	P<0.00001	/
	Auricular acupoints (42, 45)	194	SMD -2.16 [-2.70, -1.62]	P<0.00001	I^2 =^ 57%
	**Acupoint combination**				
	Cancer pain (42, 45, 56)	263	SMD -1.62 [-2.73, -0.51]	P<0.00001	I^2 =^ 93%
	sSeep disturbances and cancer pain (59)	100	MD -2.12 [-2.55, -1.69]	P<0.00001	/
	**Frequency of treatment session**				
	1/d (56, 59)	169	MD -1.37 [-2.38, 0.11]	P=0.07	I^2 =^ 95%
	2/d (45)	110	MD -0.91 [-1.05, -0.77]	P<0.00001	/
	6/d (42)	84	MD -1.08 [-1.32, -0.84]	P<0.00001	/
	**Duration time**				
	3–5 min (42)	84	MD -1.08 [-1.32, -0.84]	P<0.00001	/
	20 min (56)	69	MD -0.61 [-1.12, -0.10]	P=0.02	**/**
	30 min (59)	100	MD -2.12 [-2.55, -1.69]	P<0.00001	/
	Not mentioned (45)	110	MD -2.43 [-2.93, -1.94]	P<0.00001	/
	**TNM stage**				
	Late stage (42, 45, 59)	294	SMD -2.07 [-2.43, -1.72]	P<0.00001	I^2 =^ 34%
	Middle and late stage (56)	69	MD -0.61 [-1.12, -0.10]	P=0.02	**/**

TNM, tumor–node–metastasis.

On two EORTC QLQ-C30 measures of QOL (41, 57), patients receiving acupuncture-related treatment had remarkably higher mean scores than patients from the control group (MD 10.68, 95%CI, 4.56 to 16.81, P=0.0006, I^2^ = 51%). For the QOL measured by FACT-L, the TEAS and fire needle used by Sun Y et al. (61) and Pei WY et al. (65) worked better (MD 4.65, 95%CI, 1.67 to 7.63, P=0.002, I^2^ = 26%). For the QOL measured by FACT-LCS, a significant reduction in the FACT-LCS score was observed in the 14 participants who received active acupuncture compared with those receiving the placebo (MD 5.80, 95%CI, 4.63 to 6.97, P<0.00001). In terms of the QOL measured by LCQ, Ma HX et al. (50) concluded that the improvements in the LCQ score in the treatment group were better than the control group (MD 20.21, 95%CI, 15.61 to 24.81, P<0.00001). Based on the QOL measured by SF-36 (58), the scores of physiological function, physiological function, general health, social function, emotional intelligence, and mental health in the observation group were significantly higher than those in the control group (MD 10.36, 95%CI, 6.17 to 14.55, P<0.00001). For the QOL measured by SGRQ (68), the intervention group’s life quality scores significantly decreased (MD -31.58, 95%CI, -36.58 to -26.80, P<0.00001).

### Pain

**Figure 3:**
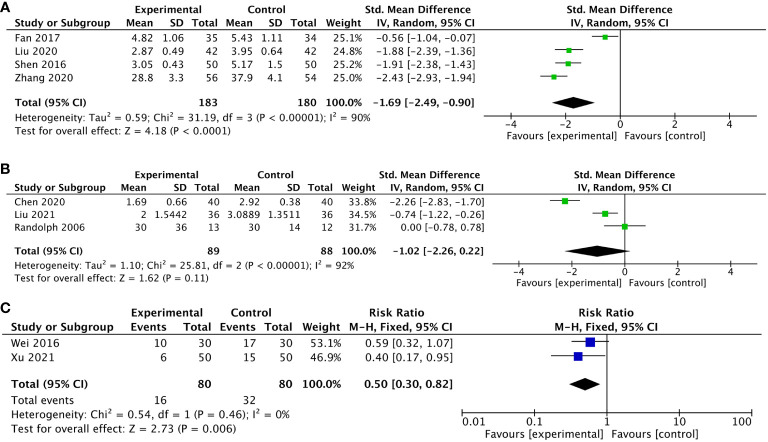
Forest plot of the pain in lung cancer patients treated with acupuncture and control. **(A)** The changes measured by the combination of the Numerical Rating Scale (NRS) score and Visual Analog Scale (VAS) pain scales in lung cancer patients with cancer pain treated with acupuncture-related intervention versus control from the baseline to the end of treatment; **(B)** the changes measured by the combination of NRS and VAS scores in lung cancer patients undergoing surgery treated with acupuncture-related intervention versus control from the baseline to the end of treatment; **(C)** the changes measured by the combination of the NRS score in lung cancer patients with cancer pain treated with acupuncture-related intervention versus control from the baseline to the end of treatment using dichotomous variables. IV, inverse variance; CI, confidence interval.

Eleven RCTs (41, 42, 45, 56–60, 62, 69, 70) reported on pain by patients, and used four pain measures including the NRS score (42, 56, 58, 59, 69, 70), VAS (45, 60, 62), and PA score in EORTC QLQ-C30 (41, 57) or BPI-R (41). Among these 11 trials, 6 trials used LI4, 4 trials used ST36, 3 trials used TF4 and AH6a, and 2 trials used BL13, PC6, and AT4. Higher scores were considered worse in all these PROs. We combined the data of two pain scales, VAS and NRS, and classified them according to different stages of the disease. We reached the conclusions that for patients of cancer pain (42, 45, 56, 59), compared with those receiving Western medicine treatment and usual care, patients receiving acupuncture-related treatment improved significantly (SMD -1.69, 95%CI, -2,49 to -0.90, P<0.0001, I^2^ = 90%). However, for patients of postoperative pain (60, 62, 69), the results showed no statistical significance (SMD -1.20, 95%CI, -2.26 to 0.22, P = 0.11, I^2^ = 92%). There were two trials (58, 70) measured by the NRS that used dichotomous variables with the patients of cancer pain, showing that the pain relief efficiency in the observation group was significantly higher than that of the control group (RR 0.50, 95%CI, 0.30 to 0.82, P=0.006, I^2^ = 0%) (**Figure 3**).

For pain measured by the PA score in EORTC QLQ-C30, Wang X et al. (41) and Wang LQ et al. (57) compared the PA scores of patients in the observation group after treatment with those in the control group, but the differences were not statistically significant in both two trials (P>0.05) (MD -3.90, 95%CI, -9.33 to 1.54, P=0.16, I^2^ = 58%). One RCT (41) using BPI-R showed that there was significant difference in the pain intensity between the observation group and the control group after treatment and between the observation group after treatment and before treatment (**Table 5**).

**Table 5:** The effect of acupuncture on the secondary outcomes compared to different comparators.

Outcome	Participants	End of treatment				Meaning of higher scores
		IV, Random, 95% CI	M-H, Fixed, 95% CI	P-value		
**1. Acupuncture vs. WM/TCM** 1.1 Pain						
NRS (56, 58, 59, 70)	329		RR 0.47 [0.29, 0.75]		P=0.001	worse
NRS + VAS (42, 45, 56, 58, 59, 69, 70)	435	SMD -1.16 [-1.55, -0.76]			P<0.00001	worse
1.2 Nausea and vomiting						
MAT (51)	160		RR 0.59 [0.41, 0.86]		P=0.006	worse
INVR (55)	96	MD -1.18 [-1.89, -0.47]			P=0.001	worse
1.3 Insomnia						
PSQI (43, 52, 59, 73)	346	MD -2.68 [-3.49, -1.86]			P<0.00001	worse
1.4 Fatigue						
PFS-R (42, 63)	246	MD -0.88 [-1.08, -0.69]			P<0.00001	worse
1.5 Anxiety and depression						
SAS (45, 59)	210	MD -4.74 [-6.66, -2.82]			P<0.00001	worse
SDS (45, 59)	210	MD -6.02 [-8.11, -3.94]			P<0.00001	worse
**2. Acupuncture vs. placebo**						
1.1 Fatigue						
BFI-C (54)	28	MD -1.40 [-1.62, -1.18]			P<0.00001	worse
**3. Acupuncture vs. sham acupuncture**						
1.1 Pain						
VAS (60, 62)	105	MD -1.08 [-1.87, -0.29			P=0.007	worse
**2. Acupuncture vs. usual care**						
1.1 Pain						
QLQ-C30 PA (41, 57)	214	MD -3.90 [-9.33, 1.54]			P=0.16	worse
BPI (41)	118	MD -3.22 [-3.66, -2.78]			P<0.00001	worse
1.2 Nausea and vomiting						
QLQ-C30 NV (41, 57)	214	MD -14.73 [-23.88, -5.59]			P=0.002	worse
1.3 Insomnia						
PSQI (71)	75	MD -6.86 [-7.59, -6.13]			P<0.00001	worse
AIS (44)	60	MD -0.17 [-1.93, 1.59]			P=0.85	worse
QLQ-C30 SL (41) 1.4 Fatigue	118	MD -14.16 [-20.91, -7.41]			P<0.00001	worse
BFI-C (67)	105		RR 0.84 [0.73, 0.97]		P=0.02	worse
PFS-R (71, 72)	195	MD -1.61 [-2.10, -1.12]			P<0.00001	worse
1.5 Constipation						
QLQ-C30 CO (41)	118	MD -12.70 [-19.52, -5.88]			P=0.0003	worse

QLQ-C30, European Organization for Research and Treatment of Cancer Quality of Life Questionnaire; NRS, Numerical Rating Scale; VAS, Visual Analog Scale; BPI-C, Brief Pain Inventory–Chinese Version; MAT, MASCC (Multinational Association of Supportive Care in Cancer) Antiemesis Tool; INVR, Index of Nausea and Vomiting and Retching; SAS, Self-Rating Anxiety Scale; SDS, Self-Rating Depression Scale; AIS, Athens Insomnia Scale; PSQI, Pittsburgh Sleep Quality Index; BFI-C, Brief Fatigue Inventory–Chinese Version; PFS-R, The Revised Piper Fatigue Scale; WM, Western medicine; TCM, traditional Chinese medicine.

Subgroup analysis showed that studies with all types of the methods above had significant effect on alleviating cancer pain. In the analysis of acupuncture technique treatment, auricular acupoint treatment showed lower heterogeneity and increased effect size on reducing cancer pain (SMD -2.16, 95%CI, -2.70 to -1.62, P<0.00001, I^2^ = 57%). In the analysis of the TNM stage, treatments in the studies of late stage showed significant improvement in patients with lung cancer–related pain and lower heterogeneity (SMD -2.07, 95%CI, -2.43 to -1.72, P<0.00001, I^2^ = 34%). In the analysis of acupoint combination, studies showed a significant effect on cancer pain reduction but could not explain the heterogeneity (SMD -1.62, 95%CI, -2.37 to -0.51, P<0.00001, I^2^ = 93%). In the analysis of the frequency of treatment session, studies showed no statistical difference at reducing cancer pain (SMD -1.37, 95%CI, -2.38 to 0.11, P = 0.07, I^2^ = 95%). In the analysis of duration time, because of the different duration time of all four studies, heterogeneity could not be explained.

After further subgroup analysis, we found that Fan LY et al. (56) was the main source of heterogeneity. This trial was focused on the effect of manual acupuncture in improving patients with lung cancer–related pain at middle and late stage; therefore, the most difference between it and other trials lied in the different TNM stage. Additionally, we performed another subgroup analyses of all the outcomes based on the different control strategies used, and the results are shown in **Tables 4** and **5**.

**Table 4:** The effect of acupuncture on the QOL compared to different comparators.

Outcome	Participants	End of treatment				Meaning of higher scores
		IV, Random or Fixed, 95% CI	M-H, Fixed, 95% CI		P-value	
**1. Acupuncture vs. WM/TCM**						
KPS (42, 45, 48–50, 53, 65, 66, 69)	792	MD 6.88 [5.88, 7.89]		P<0.00001		better
KPS (46, 64)	222		RR 1.26 [1.10, 1.44]	P=0.001		better
SF-36 (58)	100	MD 10.36 [6.17, 14.55]		P<0.00001		better
FACT-L (65)	60	MD 8.76 [2.05, 15.47]		P=0.01		better
LCQ (50)	120	MD 20.21 [15.61, 24.81]		P<0.00001		better
**2. Acupuncture vs. placebo**						
FACT-LCS (54)	28	MD 5.80 [4.63, 6.97]		P<0.00001		better
**3. Acupuncture vs. usual care**						
KPS (47, 61)	208	MD 5.93 [3.43, 8.44]		P<0.00001		better
FACT-L (61)	120	MD 3.64 [0.32, 6.96]		P=0.03		better
EORTC QLQ-C30* (41, 57)	214	MD 10.68 [4.56, 16.81]		P=0.0006		better
SGRQ (68)	60	MD -31.69 [-36.58, -26.80]		P<0.00001		worse

*Random-effect model was used for the high heterogeneity (I^2^>50%). QOL, quality of life; KPS, Karnofsky Performance Status; QLQ-C30, European Organization for Research and Treatment of Cancer Quality of Life Questionnaire; FACT-LCS, the Functional Assessment of Cancer Therapy Lung Cancer Subscale; SF-36, the MOS item short form health survey; LCQ, Leicester Cough Questionnaire; FACT-L, Functional Assessment of Cancer Therapy–Lung; WM, Western medicine; TCM, traditional Chinese medicine; SGRQ, St George’s Respiratory Questionnaire.

### Sensitivity analysis

In order to explore the stability of the results and the sources of heterogeneity in our meta-analysis, we pooled all studies for sensitivity analysis by excluding each study individually.

In terms of the cancer pain measured by the combination of VAS and NRS, there were significant changes in the outputs after excluding each study. After removing the study conducted by Fan LY et al. (56), the heterogeneity was significantly reduced and the result did not alter (SMD -2.07, 95%CI, -2.43 to -1.72, P<0.00001, I^2^ = 34%). In terms of the postoperative pain measured by the combination of VAS and NRS, the heterogeneity did not alter but the result changed to statistically significant after removing the study of Randolph et al. (60) (SMD -1.49, 95%CI, -2.98 to -0.01, P<0.05, I^2^ = 94%). After removing the study of Chen et al. (62), the heterogeneity decreased from 92% to 60% but with no statistical significance.

In terms of the sleep disturbances measured by PSQI, the heterogeneity decreased from 97% to 74% after removing the study of Yang H et al. (71).

In terms of the fatigue measured by PFS, after removing the study of Yang H et al. (71), the heterogeneity was significantly reduced to 0% and the result did not alter (MD -0.91, 95%CI, -1.10 to -0.73, P = 0.70, I^2^ = 0%).

### Meta regression

We used meta regression analysis to clarify the sources and value of heterogeneity, and to further explain the influence of variables on the combined effect. We explored heterogeneity by taking acupuncture technique, course of treatment, frequency of treatment, year of publication, country of publication, duration time, TNM stage as variables. The results showed that the duration time in the sleep disturbances measured by PSQI was the main source of heterogeneity (p=0.017) and the other results showed that the variables were insignificant under meta regression (p>0.05). **Supplementary Tables 5**–**8** in the Supplementary Material.

### Publication bias

**Figure 7:**
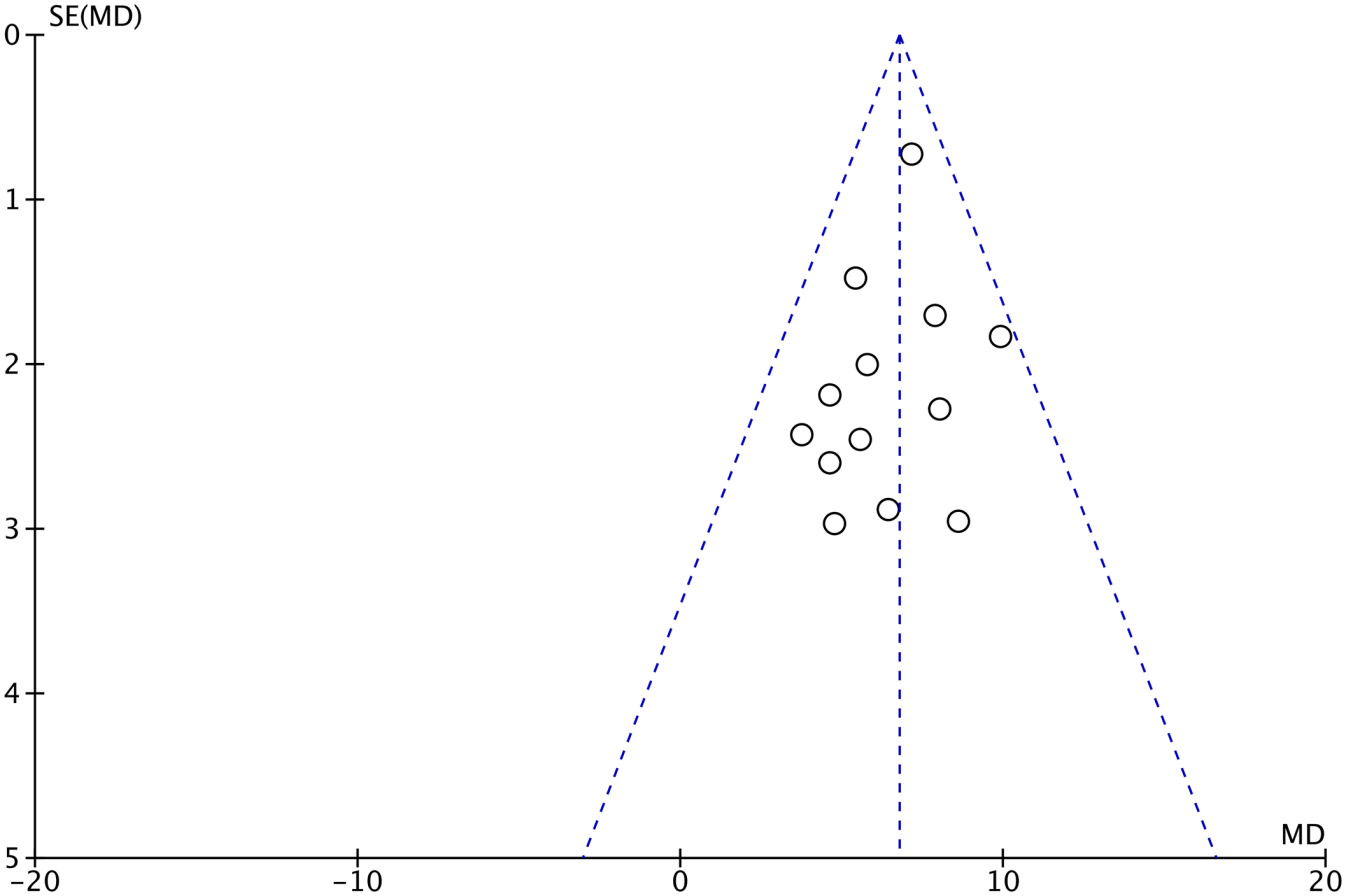
The funnel plot of quality of life measured by the Karnofsky Performance Status in lung cancer patients treated with acupuncture and control.

The funnel plot of 11 trials measured by KPS of the primary outcome QOL showed approximate symmetry (**Figure 7**). Because of the limited number of trials included for the remaining comparison measured by other PROs in the meta-analysis, funnel plots were not feasible. Therefore, we could not fully evaluate publication bias.

## Discussion

### Interpretation of the results

This study, including 33 RCTs, showed that acupuncture had a marked beneficial effect on improving PROs in different dimensions and different cancer stages or conditions. Compared with control groups such as usual care and pharmacotherapy, using acupuncture alone or in combination with other treatments can effectively relieve PROs, including postoperative chronic pain, and reduce anxiety and depression, so as to improve their QOL, which has a certain clinical application value.

Among 33 trials, 15 trials used ST36, and 7 trials used BL13, LI4, and PC6. One trial used only one acupoint (ST36) and five trials used two acupoints, showing significant efficacy in patients with lung cancer. It followed that ST36 can tonify qi and/or blood deficiency, increases stamina and energy, and it is the most important point to promote general wellness. Auricular acupoints were used in 7 of the 33 trials, among which TF4 was used in 6 trials, AT4 was used in 5 trials, and AH6a was used in 4 trials. It suggested that AT4 was a reference point for the diagnosis of nervous system diseases, tumors, insomnia, lethargy, and other diseases, which was of great importance for the diagnosis of malignant tumors (75). We also divided 33 trials into 4 different types to observe the commonness of acupoint selection. Of all five surgery-related trials, three used LI4 and two used ST36, SP6, and SP10. Of all 12 chemotherapy-related trials, 8 used ST36 and 4 used RN12 and PC6. One radiotherapy-related trial used auricular acupoints. The remaining trials were about lung cancer itself, and six of them used BL13 and five used LI4 and ST36. Thus, ST36 was useful in all types of trials except for the radiotherapy trials because of the selection of auricular acupoints. This study also provides a reference for the selection of effective acupoints for remedying various disorders in the PRO outcome of lung cancer patients, including the QOL, pain, nausea and vomiting, sleep disturbances, fatigue, anxiety and depression, constipation, and side effect. For example, sleep disturbance is a prominent concern in lung cancer patients, which is linked to worse prognosis and a poorer QOL (76, 77). In our study, the following acupoints were selected for sleep disorders in these trails: TF4, AT4, ST36, AH6a, LI4, BL13. However, one study found that the top 10 most frequently selected acupoints for sleep disorders were HT7, SP6, PC6, KI1, GV20, EM5, EX-HN3, EX-HN16, KI3, and MA-TF1 and also suggested that the acupoints of EX-HN3, EX-HN16, GV20 integrated with HT7, KI1, PC6 are the kernel acupoint combination in the field of acupuncture therapies for sleep disorders based on an association rule analysis (78). These acupoints are completely inconsistent with our findings. The selection of acupoints during treatment is one of the main factors affecting the efficacy of acupuncture treatment. Thus, according to the different dimensions of the QOL of lung cancer patients, optimizing and screening acupoints should be the focus of future studies.

Among 33 trials, only five trials involved follow-up, and the longest one lasted only a month. The sustained and long-term effect of acupuncture on the RPOs of patients with lung cancer is unknown since most included trials ranged in duration from 4 to 8 weeks.

Eight trials reported side effects but did not state in detail whether they were caused by acupuncture or chemotherapy. However, all the trials reported that the side effects of involved acupuncture groups were lower than those of the control group. This indicated that conventional Western medicine combined with acupuncture had the advantage of reducing toxicity and increasing effects in the treatment of lung cancer, reflecting the advantages of acupuncture in lung cancer such as safety, effectiveness, urgency, acceptability, and applicability.

Eighteen trials were about the treatment of chemotherapy-induced lung cancer-related symptoms by acupuncture, and five were about postoperative. It shows that acupuncture has more opportunities to be used in the above two situations, reflecting the advantages of acupuncture. The results showed that the scores with PROs of acupuncture on patients were also higher than that of the control group, indicating that acupuncture had a significant effect on both the QOL and other patients’ self-conscious symptoms. Acupuncture could improve the PRO of lung cancer patients at different TNM stages and under different treatments of disease.

### Exploration of heterogeneity from the patient-reported outcomes

Among the 33 included RCTs, the heterogeneity obtained by approximately half of the trials was low, which meant maybe unimportant (less than 40% according to the Cochrane Handbook for Systematic Reviews of Interventions), while obvious heterogeneity was shown in several results of meta. Among them, heterogeneity 58% and 81% was derived from two secondary outcomes in pain and insomnia. The reason for the high heterogeneity of these two groups of data after the combination was that we highly suspected the existence of problems in the original data from the same trial (57). Wang LQ et al. (57) stated in the study that two secondary outcomes QLQ-C30 NV and FA in the observation group were significantly decreased compared with the control group (P<0.05), but the data showed totally opposite results that the scores of the observation group were dramatically higher than the control group (higher scores were considered worse in these two PROs). Judging from the data, the quality of this study is debatable and should be removed. However, the primary outcome in this study was the QOL of patients with the QLQ-C30, so we chose to retain the data in this study.

Heterogeneity 90% and 92% came from the cancer pain and postoperative pain measured by the combination of VAS and NRS. After subgroup and sensitivity analysis, we found the heterogeneity based on Fan LY et al. (56) in the patients of cancer pain. This may be due to the different TNM stage of patients in this trial compared with others. However, among patients of postoperative pain, Randolph et al. (60) stated that there was a trend for lower average VAS pain scores from postoperative day 2 to day 6 in the EA group, but this did not reach statistical significance. This is most likely secondary to the error from the small sample size. When we removed the study of Chen et al., the heterogeneity decreased to 60%. The major difference between this group was that patients in this trial received TEAS for 30 min before anesthetic induction and continuous stimulation throughout the whole surgical procedure.

Heterogeneity 97% from PSQI was based on Yang H et al. (71) and Kou XW et al. (52). Heterogeneity 94% from PFS was based on the same trial, Yang H et al. (71). After meta regression, we found that the duration time in the sleep disturbances measured by PSQI was the main source of heterogeneity (p=0.017), and the heterogeneity decreased from 97% to 30% after removing these two studies. The remaining two trials (59, 73) had the same duration time of treatment due to their similar interventions, which was treated by warm acupuncture and EA. Therefore, the heterogeneity was lower compared with the other two trials using thunder-fire moxibustion and acupoint application, respectively. The male patients included in Yang H et al. (71) accounted for 81.3%, which was the four times the number of women. Moreover, the majority of patients are in the stage of I and II, which may lead to the better effect of treatment. The most significant difference was evidenced by the type of the intervention, which was the only one among the 33 included trials that used thunder-fire moxibustion as the observation group for treatment in lung cancer chemotherapy patients. A study showed that thunder-fire moxibustion has anti-inflammatory effects (79). Currently, the mechanism of cancer-related fatigue is the inflammatory hypothesis that has attracted the most attention of scholars (80). The authors suggested that thunder-fire moxibustion could relieve the fatigue symptom and improve the QOL of lung cancer patients.

We combined the trials of QOL in the different TNM stages or different stages of disease and in different comparators including WM/TCM, usual care, and placebo. The results showed that the heterogeneity was low and even no heterogeneity. After subgroup and sensitivity analysis, we found that heterogeneity varied within a small range, which did not influence the stability of results. We analyzed the possible reasons as follows: first, we strictly formulated the inclusion and exclusion criteria, so the included articles were of high homogeneity; second, this represented no statistical heterogeneity, which may exist in clinical and methodological heterogeneity; third, there were still individual differences in patients’ stages of disease.

### Risk of bias

First, for selection bias. 29 of the 33 RCTS included in this study did not mention allocation concealment. During the treatment, due to the particularity of acupuncture, it is obviously impractical and difficult to blind the experimenter as well as the patients. However, for efficacy assessors and statistical analysts, blinding can be performed to minimize possible detection bias, and no one except four studies took this factor into account. Furthermore, some PROs such as the QOL instruments are often used as non-primary outcomes in studies; authors often do not present detailed numerical or exact results (as mentioned above) or even only provide bar chart results without extracting specific numbers for statistics, which may lead to publication bias.

### Clinical implications

In this study, different acupuncture interventions were used as study objects for evaluation, and it was found that manual acupuncture, acupoint application, and auricular point were most frequently used in clinical practice. Acupoint application and the auricular point had good curative effects and, meanwhile, could be treated anytime and anywhere, which was worthy of clinical application and promotion.

The advantage of our study is that we only focused on PROs with lung cancer patients, while other studies used scales that were not PROs or not just PROs to assess the treating effects of lung cancer such as the index of immunomodulation. Compared with other studies of the same type, we not only paid attention to the change of QOL with lung cancer patients by acupuncture but also to the change of lung cancer–related symptoms, which made the results more comprehensive and effective.

In addition, some trials were of poor quality and were not reported according to the reporting specifications of CONSORT (81). For instance, the lack of follow-up of PROs and side effect reports of patients were not conducive to the judgment of the QOL of lung cancer patients. We believed that the long-term follow-up of patients to assess their subsequent PRO is clinically significant. We suggested that we should pay more attention to PROs clinically in order to judge the most real situation of the patient fully and comprehensively. Moreover, the lack of research reports on the qualifications of acupuncturists indicates a lack of attention to them. Randomized controlled trials have proven that acupuncturists with different qualifications have different therapeutic effects on acupuncture (82), so we suggest that studies at home and abroad should focus on relevant information reports.

Research on acupuncture for cancer pain has been proven. Acupuncture and/or acupressure were significantly associated with reduced pain in cancer patients compared with sham surgery controls, according to a study published in JAMA Oncology (83). In other words, acupuncture is effective in reducing cancer pain and the use of opioid painkillers. With the improvement of early screening technology and treatment of lung cancer, the survival rate of lung cancer patients will be improved, and the advantages of acupuncture in early lung cancer as well as in surgery, radiotherapy and chemotherapy, immunotherapy, and other situations will be increasingly obvious. Additionally, the synergistic optimization effect of acupuncture and targeted therapy needs further research in the future.

On this basis, the pursuit of the treatment concept of prolonging the life of lung cancer patients and constantly improving the QOL of patients with lung cancer has become the research direction of more and more researchers. In this study, we evaluated the evidence from published RCTs and found that acupuncture for lung cancer and its treatment-related symptoms has the advantages of high acceptability and safety, as well as good effects on PROs with lung cancer patients. Among the main outcome measures included in this study, the most frequently used scales were KPS, QLQ-C30, and FACT-L. We hope to popularize the PRO-scale clinical trials of acupuncture for LC in the future in order to focus on the effect of acupuncture in patients with lung cancer on their PROs, mainly in the aspect of QOL.

### Limitation of evidence

Considering that various designs and techniques of acupuncture were included in this study, there is a potential risk of heterogeneity in the results. A total of 15 different interventions were included in this study, including a combination of two or three acupuncture-related interventions or just one intervention. Different experimental interventions are different in the frequency and cycle of treatment. For the same interventions, there are slight differences in therapeutic efficacy due to different acupuncturists. Second, since a rigorous search and screening strategy was used to obtain available studies, the sample size of the included studies was not particularly large. Controversial academic dissertations that have not been peer-reviewed, as well as trials that have been found to reuse data during data extraction, were excluded, resulting in only 27 trials in Chinese and 6 trials in English. Due to the inclusion of trials, which were mostly published in Asia, there are bias and limited generalizability of the conclusions to some extent. Additionally, due to the limited sample size included and the low quality of the original study, Grading of Recommendations, Assessment, Development and Evaluations (GRADE) was not used to construct the system of evidence. Furthermore, considering that acupuncture may be applied to the various stages of lung cancer and situations, we did not restrict the inclusion of specific population conditions. The age of the population we included was concentrated around 50 years old and was not representative of all adults, especially the elderly, which was mainly limited by the age of the population in the original studies and the epidemiological characteristics of lung cancer.

## Conclusion

Our study indicates that acupuncture therapies is a promising intervention in promoting PROs in lung cancer patients with all stages and regardless of postsurgery or postchemotherapy. Acupuncture should be recommended as a beneficial alternative strategy for lung cancer patients on clinic. High-quality, large-sample, multicenter original RCTs of acupuncture that focus on PROs are needed in the future.

## Data availability statement

The original contributions presented in the study are included in the article/**Supplementary Material**. Further inquiries can be directed to the corresponding authors.

## Author contributions

KW and JZ conceived and designed the study. KW, XW, and ZX searched the databases and screened the articles. ZX, XW, and ZY were involved in data extraction and the assessment of methodological quality. ZX and XW analyzed the data. All the authors contributed to the composition of the manuscript. All the authors have checked manuscripts and approved the publication of the study.

## Funding

This study was supported by grants from the Project BEBPC-TCM (2019XZZX-ZJ0011); Shanghai Clinical Research Center for Acupuncture and Moxibustion Accelerating (20MC1920500); Clinical Key Specialty Construction Foundation of Shanghai (shslczdzk04701).

## Acknowledgments

We thank all authors and participants in this study.

## Conflict of interest

The authors declare that the research was conducted in the absence of any commercial or financial relationships that could be construed as a potential conflict of interest.

## Publisher’s note

All claims expressed in this article are solely those of the authors and do not necessarily represent those of their affiliated organizations, or those of the publisher, the editors and the reviewers. Any product that may be evaluated in this article, or claim that may be made by its manufacturer, is not guaranteed or endorsed by the publisher.
