# Supplementary material for: Acupuncture for adult lung cancer of patient-reported outcomes: A systematic review and meta-analysis
Source: Front Oncol. 2022 Sep 2;12:921151. doi: 10.3389/fonc.2022.921151 (PMC9479629; doi:10.3389/fonc.2022.921151)
Supplement: Supplementary file 1 [file Table_1.docx]

| **Supplementary Table 1** **\|** The search strategy for English databases. | | |
| --- | --- | --- |
| **Database** | **Order** | **Strategy** |
| Cochrane Library | #1 | MeSH descriptor: [Lung Neoplasms] explode all trees |
|  | #2 | lung |
|  | #3 | cancer OR tumor OR carcinoma OR neoplasm |
|  | #4 | #2 AND #3 |
|  | #5 | #1 OR #4 |
|  | #6 | MeSH descriptor: [Acupuncture] explode all trees |
|  | #7 | MeSH descriptor: [Acupuncture Therapy] explode all trees |
|  | #8 | MeSH descriptor: [Auriculotherapy] explode all trees |
|  | #9 | MeSH descriptor: [Cupping Therapy] explode all trees |
|  | #10 | MeSH descriptor: [Transcutaneous Electrical Acupoint Stimulation] explode all trees |
|  | #11 | MeSH descriptor: [Acupuncture Points] explode all trees |
|  | #12 | MeSH descriptor: [Acupuncture, Ear] explode all trees |
|  | #13 | MeSH descriptor: [Moxibustion] explode all trees |
|  | #14 | (Acupuncture OR Electroacupuncture OR Acupressure OR Auriculotherapy OR Moxibustion OR Cupping therapy) OR (Dry needling) OR (Transcutaneous Electric Nerve Stimulation) OR (Acupuncture Points) OR (Acupuncture Ear) |
|  | #15 | #6 OR #7 OR #8 OR #9 OR #10 OR #11 OR #12 OR #13 OR #14 |
|  | #16 | random OR randomization OR randomized OR randomised OR randomly |
|  | #17 | #5 AND #15 AND #16 in Trials |
| EMBASE | #29 | #9 AND #22 AND #28 |
|  | #28 | #23 OR #24 OR #25 OR #26 OR #27 |
|  | #27 | randomly' |
|  | #26 | randomised' |
|  | #25 | randomized' |
|  | #24 | randomization' |
|  | #23 | random' |
|  | #22 | #10 OR #21 |
|  | #21 | #11 OR #12 OR #13 OR #14 OR #15 OR #16 OR #17 OR #18 OR #19 OR #20 |
|  | #20 | Moxibustion':ab,kw,ti |
|  | #19 | Acupuncture Ear ':ab,kw,ti |
|  | #18 | Acupuncture Points ':ab,kw,ti |
|  | #17 | Transcutaneous Electric Nerve Stimulation':ab,kw,ti |
|  | #16 | Dry needling':ab,kw,ti |
|  | #15 | Cupping therapy':ab,kw,ti |
|  | #14 | auriculotherapy':ab,kw,ti |
|  | #13 | acupressure':ab,kw,ti |
|  | #12 | electroacupuncture':ab,kw,ti |
|  | #11 | acupuncture':ab,kw,ti |
|  | #10 | 'acupuncture'/exp |
|  | #9 | #1 OR #8 |
|  | #8 | #2 AND #7 |
|  | #7 | #3 OR #4 OR #5 OR #6 |
|  | #6 | carcinoma':ab,kw,ti |
|  | #5 | neoplasm':ab,kw,ti |
|  | #4 | cancer':ab,kw,ti |
|  | #3 | tumor':ab,kw,ti |
|  | #2 | Lung':ab,kw,ti |
|  | #1 | Lung Neoplasms '/exp |
| Pubmed |  | (("Lung Neoplasms"[Mesh])) OR "Carcinoma, Non-Small-Cell Lung"[Mesh] AND  (((((("Acupuncture"[Mesh] OR "Acupuncture Therapy"[Mesh] OR "Acupuncture, Ear"[Mesh] OR "Acupuncture Points"[Mesh]) OR "Moxibustion"[Mesh]) OR "Electroacupuncture"[Mesh]) OR "Transcutaneous Electrical Acupoint Stimulation"[Mesh]) OR "Auriculotherapy"[Mesh]) OR "Dry Needling"[Mesh]) OR "Cupping Therapy"[Mesh] Filters: Randomized Controlled Trial Sort by: Publication Date |
| Web of Science | #1 | **(TS=(Lung Neoplasms)) OR TS=(Carcinoma, Non-Small-Cell Lung)** |
|  | #2 | **(((((((((TS=(Acupuncture)) OR TS=(Acupuncture Therapy)) OR TS= (Acupuncture, Ear)) OR TS=(Acupuncture Points)) OR TS=(Moxibustion)) OR TS=(Electroacupuncture)) OR TS=(Transcutaneous Electrical Acupoint Stimulation)) OR TS=(Auriculotherapy)) OR TS=(Dry Needling)) OR TS=(Cupping Therapy)** |
|  | #3 | **((((TS=(random)) OR TS=(randomization)) OR TS=(randomized)) OR TS=(randomised)) OR TS=(randomly)** |
|  | #4 | **TS=(Trials)** |
|  | #5 | **#1 AND #2 AND #3 AND #4** |
