# Supplementary material for: Acupuncture for adult lung cancer of patient-reported outcomes: A systematic review and meta-analysis
Source: Front Oncol. 2022 Sep 2;12:921151. doi: 10.3389/fonc.2022.921151 (PMC9479629; doi:10.3389/fonc.2022.921151)
Supplement: Supplementary file 2 [file Table_2.docx]

| **Supplementary Table 2** **\|** The search strategy for Chinese databases. | |  |
| --- | --- | --- |
| **Database** | **Strategy** |  |
| CNKI | (SU=’肺癌’ OR SU=’肺肿瘤’ OR SU=’非小细胞肺癌’ ) AND (SU=’针灸疗法’ OR SU=’针灸’ OR SU=’针刺’ OR SU=’艾灸’ OR SU=’电针’ OR SU=’头针’ OR SU=’耳针’ OR SU=’耳穴’ OR SU=’三棱针’ OR SU=’腹针’ OR SU=’梅花针’ OR SU=’火针’ OR SU=’拔罐’ OR SU=’皮肤针’ OR SU=’皮内针’ OR SU=’穴位敷贴’ OR SU=’穴位埋线’ OR SU=’穴位注射’ OR SU=’经皮穴位电刺激’) AND (TKA=’随机’) |  |
| Wangfang Data | ((((((((((主题=穴位敷贴) OR 主题=穴位埋线) OR 主题=穴位注射) OR 主题=经皮穴位电刺激)) OR ((((((主题=腹针) OR 主题=梅花针) OR 主题=火针) OR 主题=拔罐) OR 主题=皮肤针))) OR ((((((主题=头针) OR 主题=耳针) OR 主题=耳穴) OR 主题=三棱针) OR 主题=皮内针))) OR ((((((主题=针灸疗法) OR 主题=针灸) OR 主题=针刺) OR 主题=艾灸) OR 主题=电针)))) AND ((((题名=肺癌) OR 题名=肺肿瘤) OR 题名=非小细胞肺癌))) AND (题名或关键词=随机) |  |
| VIP | T=(肺癌 OR 肺肿瘤 OR 小细胞肺癌 OR 非小细胞肺癌) AND M=(针灸疗法 OR 针灸 OR 针刺 OR 艾灸 OR 电针 OR 头针 OR 耳针 OR 耳穴 OR 三棱针 OR 腹针 OR 梅花针 OR 火针 OR 拔罐 OR 皮肤针 OR 皮内针 OR 穴位敷贴 OR 穴位埋线 OR 穴位注射 OR 经皮穴位电刺激) AND M=(随机) |  |
| Sinomed | ("随机对照试验"[不加权:扩展]) AND ((((((((("温针疗法"[不加权:扩展] OR "针灸疗法"[不加权:扩展] OR "处方, 针灸"[不加权:扩展] OR "太乙针灸"[不加权:扩展] OR "针灸研究"[不加权:扩展] OR "针灸效应"[不加权:扩展]) OR "针刺穴位"[不加权:扩展] OR "穴, 阿是"[不加权:扩展]) OR "电针"[不加权:扩展]) OR "灸法"[不加权:扩展]) OR "头针"[不加权:扩展]) OR "耳针"[不加权:扩展]) OR "刺血疗法"[不加权:扩展]) OR "穴位贴敷法"[不加权:扩展]) OR "穴位埋线结扎"[不加权:扩展]) AND ("肺肿瘤"[不加权:扩展] OR "肺肿瘤"[不加权:扩展] OR "小细胞肺癌"[不加权:扩展] OR "癌, 非小细胞肺"[不加权:扩展] OR "癌, 非小细胞肺"[不加权:扩展]) |  |
