# Supplementary material for: Acupuncture for adult lung cancer of patient-reported outcomes: A systematic review and meta-analysis
Source: Front Oncol. 2022 Sep 2;12:921151. doi: 10.3389/fonc.2022.921151 (PMC9479629; doi:10.3389/fonc.2022.921151)
Supplement: Supplementary file 3 [file Table_3.docx]

| **Supplementary Table 3** **\|** The Characteristics of the Included Trials. | | | | |  |  |  |  |
| --- | --- | --- | --- | --- | --- | --- | --- | --- |
| **Author (Year)** | **Condition/Stage** | **Age** | **Randomization** | **Intervention (n)** | | **Outcome** | | |
|  |  | **I/C** |  | **Control (n)** | | **Primary outcome** | **Secondary outcome** | |
| Luo et al.  (2022) [47] | Patients with anorexia associated with advanced lung cancer/Ⅳ stage | I: 61.14±5.31 | Random number | I: auricular points plaster therapy + nutritional support (n=44) | | KPS | **/** | |
|  |  | C: 60.85±5.29 |  | C: nutritional support (n=44) | |  |  |  |
| Kou et al.  (2021) [52] | Cancer-related insomnia/Ⅰ-Ⅱ stage | I: 66.75±3.72 | Random number table | I: Acupoint Application + WM (n=35) | | / | PSQI | |
|  |  | C: 67.47±3.23 |  | C: WM (n=35) | |  |  |  |
| Bai et al.  (2021) [73] | Sleep disturbance after chemotherapy for lung cancer | I: 50.1±11.4 | Random number table | I: Mongolia traditional medicine Sugemule-3 + Mongolian medicine warm acupuncture (n=30) | | **/** | PSQI | |
|  |  | C: 52.6±12.4 |  | C: WM (n=30) | |  |  |  |
| Liu et al.  (2021) [69] | Cancer pain/post-surgery | I: 50.19±6.93 | Regional random grouping | I: Plum-blossom needle tapping + WM (n=36) | | KPS | NRS  BPI | |
|  |  | C: 50.81±7.82 |  | C: WM (n=36) | |  |  |  |
| Yang et al. (2021) [71] | Fatigue, insomnia/Undergoing chemotherapy | I: 47.96±8.04 | Random number table | I: Thunder-fire moxibustion + Usual care (n=42) | | / | PSQI  PFS⁃R | |
|  |  | C: 47.65±7.67 |  | C: Usual care (n=42) | |  |  |  |
| Xu et al.  (2021) [58] | Cancer pain | I: 53.12±2.54 | Random number table | I: Moxibustion + WM (n=50) | | SF-36 | NRS | |
|  |  | C: 55.30±2.98 |  | C: WM (n=50) | |  |  |  |
| Dogan et al.  (2020) [68] | Patients in the unit who were receiving chemotherapy and had experienced dyspnea/Ⅱ-Ⅳ stage | I: 59.01±8.11 | computerized randomization | I: Acupressure (n=29) | | SGRQ | / | |
|  |  | C: 63.09±7.99 |  | C: Usual care (n=31) | |  |  |  |
| Cheng et al. (2020) [55] | CRNV | I_1_: 59.64±6  I_2_: 58.73±9 | Random number produced by SPSS (20.0) | I_1_: PRG (n=32)  I_2_: POG (n=34) | | / | INVR | |
|  |  | C: 60.12±8 |  | C: WM (n=34) | |  |  |  |
| Chen et al. (2020) [62] | Patients undergoing VATS pulmonary resection/Early-stage lung cancer | I: 56.0±3.7 | Computer based sample randomization | I: TEAS (n=40) | | / | VAS | |
|  |  | C: 55.8±3.2 |  | C: Sham-TEAS (n=40) | |  |  |  |
| Yu  (2020) [48] | Advanced lung cancer and pleural effusion | I: 62.21±5.36 | Random number table | I: Chinese herbal application + Moxibustion + Injection of cisplatin + WM (n=41) | | KPS | / | |
|  |  | C: 61.02±4.96 |  | C: WM (n=41) | |  |  |  |
| Sun et al.  (2020) [61] | Post-surgery with venous thrombosis/Ⅰ, Ⅱ, Ⅲa stage | I: 57±8 | Random number produced by SPSS (22.0) | I: TEAS + Rehabilitation training (n=40) | | KPS  FACT-L | / | |
|  |  | C_1_: 59±8  C_2_: 57±12 |  | C_1_: Usual care (n=40)  C_2_: Rehabilitation training (n=40) | |  |  |  |
| Zhang et al. (2020) [45] | Ⅲb, Ⅳ stage | I: 68.67±8.24 | Random number table | I: Auricular + Point application + WM (n=56) | | KPS | SAS  SDS | |
|  |  | C: 67.43±8.65 |  | C: WM (n=54) | |  |  |  |
| Wang (2020) [41] | Cancer pain/all stage | I: 45.54±4.64 | Random number table | I: Acupuncture auricular + Usual care (n=59) | | QLQ-C30 | QLQ-C30 PA, NV, FA, SL, CO | |
|  |  | C: 46.27± 3.21 |  | C: Usual care (n=59) | |  |  |  |
| Liu et al.  (2020) [42] | Cancer-related pain and fatigue/Ⅲ, Ⅳ stage | I: 67.27±6.12 | Random number table | I: Ear buried seeds + WM (n=42) | | KPS | NRS | |
|  |  | C: 66.94±5.87 |  | C: WM (n=42) | |  |  |  |
| Ma et al.  (2019) [50] | Cough and asthma patients/Middle and late stage | I: 59.31±9.16 | Random number table | I: Acupoint application + TCM + WM (n=60) | | KPS  LCQ | / | |
|  |  | C: 59.73±9.08 |  | C: WM (n=60) | |  |  |  |
| Zhou et al. (2019) [51] | Patients with nausea and vomiting induced by chemotherapy | I: 55.83±7.57 | Random number table | I: Acupoint application + EA + WM (n=80) | | / | MAT | |
|  |  | C: 53.79±8.17 |  | C: WM (n=80) | |  |  |  |
| Guo et al. (2019) [43] | Patients with insomnia induced by chemotherapy | I_1_: 57.13±8.21  I_2_: 58.37±7.87  I_3_: 55.27±9.14 | Random number table | I_1_: Auricular acupoint (n=30)  I_2_: Moxibustion (n=30)  I_3_: Auricular acupoint + Moxibustion (n=30) | | / | PSQI | |
|  |  | C: 58.54±8.29 |  | C: WM (n=26) | |  |  |  |
| Wang et al. (2019) [53] | CRNV | I_1_: 53±6  I_2_: 55±7 | Random number table | I_1_: Acupuncture + WM 30 min before chemotherapy (n=49)  I_2_: WM before chemotherapy + Acupuncture 30 min after chemotherapy (n=44) | | KPS | / | |
|  |  | C: 53±7 |  | C: WM 30 min before chemotherapy (n=47) | |  |  |  |
| Yin  (2019) [49] | CRNV | I: 51.43±10.29 | Random number table | I: Acupoint application + Acupoint injection + Low frequency pulse + WM (n=32) | | KPS | / | |
|  |  | C: 52.97±9.76 |  | C: WM (n=32) | |  |  |  |
| Deng et al. (2019) [64] | Patients with advanced NSCLC in chemotherapy /Ⅲa, Ⅲb, Ⅳ stage | I: 66.12±5.36 | Random number table | I: Acupoint injection + WM (n=40) | | KPS | / | |
|  |  | C: 66.54±5.87 |  | C: WM (n=40) | |  |  |  |
| Hou et al. (2017) [63] | Patients with fatigue induced by chemotherapy | I: 58.06±8.42 | Lottery | I: TEAS (n=59) | | / | RPFS | |
|  |  | C_1_: 54.44±10.48  C_2_: 58.27±10.57 |  | C_1_: Sham TEAS (n=52)  C_2_: WM (n=58) | |  |  |  |
| Cheng et al. (2017) [54] | Fatigue | I: 58±5.2 | Random number produced by computer | I: Acupuncture (n=14) | | FACT-LCS | BFI-C | |
|  |  | C: 62±4.3 |  | C: Acupuncture + placebo (n=14) | |  |  |  |
| Lu et al.  (2017) [44] | Patients with fatigue induced by chemotherapy | I: 42-76 | Random number table | I: Ear buried seeds + Usual care (n=30) | | / | AIS | |
|  |  | C: 36-75 |  | C: Usual care (n=30) | |  |  |  |
| Fan et al.  (2017) [56] | Fatigue/Advanced lung cancer | I: 58.42±9.64 | Random number table | I: Acupuncture + WM (n=35) | | / | NRS | |
|  |  | C: 55.39±8.99 |  | C: Usual care (n=34) | |  |  |  |
| Wang et al. (2017) [57] | Patients with lung cancer after radical operation | I: 46.2±11.4 | Random number table | I: Moxibustion + Usual care n=48) | | QLQ-C30 | QLQ-C30 PA, NV, FA | |
|  |  | C: 48.4±10.7 |  | C: Usual care (n=48) | |  |  |  |
| Pei et al.  (2017) [65] | Patients with chemotherapy of NSCLC/Ⅲa, Ⅲb, Ⅳ stage | I: 59±9 | Random number table | I: Fire needle + WM (n=30) | | KPS  FACT-L | / | |
|  |  | C: 60±9 |  | C: WM (n=30) | |  |  |  |
| Ou et al.  (2016) [46] | Ⅲb, Ⅳ stage | I: 66.84 ± 8.57 | Random number table | I: Acupuncture + Auricular acupoint + Point application (n=71) | | KPS | / | |
|  |  | C: 66.84 ± 8.57 |  | C: WM + TCM (n=71) | |  |  |  |
| Wei et al. (2016) [70] | Cancer pain | I:/ | Random number produced by computer | I: Catgut-embedding therapy +WM (n=30) | | / | NRS | |
|  |  | C:/ |  | C: WM (n=30) | |  |  |  |
| Wu et al.  (2016) [72] | Fatigue /Ⅲ, Ⅳ stage | I: 52.6 ± 6.2 | Random number table | I: Thermal moxibustion + Usual care (n=60) | | / | RFPS | |
|  |  | C: 53.3 ± 5.6 |  | C: Usual care (n=60) | |  |  |  |
| Liu  (2016) [67] | Fatigue | I:/ | Lottery | I: Acupressure + Usual care (n=53) | | / | BFI-C | |
|  |  | C:/ |  | C: Usual care (n=52) | |  |  |  |
| Shen et al. (2016) [59] | Cancer-related pain and insomnia/Ⅲb, Ⅳ stage | I: 54.93±14.45 | Random number table | I: EA + WM (n=50) | | / | NRS  SAS | PSQI  SDS |
|  |  | C: 58.09±12.11 |  | C: WM (n=50) | |  |  |  |
| Li et al.  (2014) [66] | Ⅲa, Ⅲb, Ⅳ stage | I: 55.5±11.2 | Random number table | I: Fire needle + WM (n=30) | | KPS | / | |
|  |  | C: 52.4±12.3 |  | C: WM (n=30) | |  |  |  |
| Randolph et al. (2006) | Cancer pain/Post thoracotomy | I: 64.6±8.0 | Random number produced by computer | I: EA (n=13) | | / | VAS | |
|  |  | C: 64.5±8.5 |  | C: SA (n=12) | |  |  |  |

Abbreviations: QOL, quality of life; CRNV, cancer-related nausea and vomiting; NSCLC, non-small cell lung cancer; WM, Western medicine interventions; TEAS, transcutaneous electrical acupoint stimulation; EA, Electroacupuncture; SA, sham acupuncture; CRNV, chemotherapy related nausea and vomiting; VATS, video assisted thoracic surgery; PRG, pre-chemotherapy acupuncture group; POG, post-chemotherapy acupuncture group; KPS, Karnofsky Performance Status; QLQ-C30, European Organization for Research and Treatment of Cancer Quality of Life Questionnaire; FACT-LCS, the Functional Assessment of Cancer Therapy Lung Cancer Subscale; SF-36, the MOS item short from health survey; LCQ, Leicester Cough Questionnaire; FACT-L, Functional Assessment of Cancer Therapy-Lung; NRS, Numerical Rating Scale; VAS, Visual Analogue Scale; BPI-C, Brief Pain Inventory-Chinese Version; MAT, MASCC (Multinational Association of Supportive Care in Cancer) Antiemesis; INVR, Index of Nausea and Vomiting and Retching; PSQI, Pittsburgh Sleep Quality Index; AIS, Athens Insomnia Scale; SAS, Self-Rating Anxiety Scale; SDS, Self-Rating Depression Scale; BFI-C, Brief Fatigue Inventory-Chinese Version; PFS-R, The Revised Piper Fatigue Scale; SGRQ, St George’s Respiratory Questionnaire.
