# Supplementary material for: Acupuncture for adult lung cancer of patient-reported outcomes: A systematic review and meta-analysis
Source: Front Oncol. 2022 Sep 2;12:921151. doi: 10.3389/fonc.2022.921151 (PMC9479629; doi:10.3389/fonc.2022.921151)
Supplement: Supplementary file 4 [file Table_4.docx]

| **Supplementary Table 4** **\|** Acupuncture detail of included trials | | | | | |  |
| --- | --- | --- | --- | --- | --- | --- |
| **Author Main Acupoint** | | **Course** | **Intervention time point** | **Results** | **Follow up** | |
| Luo et al.  (2022) [47] | TF4, CO4, CO13 | 4-6/D; 12D | The whole course of treatment | The increase range of KPS score in observation group was more obvious than that of the control group(P<0.05). | | NA |
| Kou et al.  (2021) [52] | BL15, PC6, SP6, HT7 | 1/D; 30D | The whole course of treatment | The scores of all indexes of the PSQI were significantly lower (P<0.05). | | NA |
| Bai et al.  (2021) [73] | DU20, DU9, DU14 | 1/D; 15D | The whole course of treatment | The daytime functional scores were lower than those of the control group (P<0.05), and there were no significant differences in other scores (P>0.05). | | NA |
| Liu et al.  (2021) [69] | Ashi-points | 2/W; 4W | The whole course of treatment | The KPS score was higher and the NRS score was lower (P<0.05). | | NA |
| Yang et al.  (2021) [71] | RN12, RN6, RN8, ST36, BL13, BL20 | 1/D; 2W | The whole course of treatment | The PFS-R and PSQI score were lower (P<0.05). | | 14 D |
| Xu et al.  (2021) [58] | BL13, LU11, L14, Ashi-points | 2/D, 10D | The whole course of treatment | The SF-36 score was higher (P<0.05). | | NA |
| Dogan et al.  (2020) [68] | LU1, LU10, PC6 | 2/D, 4W | The whole course of treatment | The intervention group’s SGRQ scores significantly decreased (P<0.05). | | NA |
| Cheng et al.  (2020) [55] | PC6, ST36 | 1/D, 5D | ①30 min before chemotherapy  ②30 min after chemotherapy | There was a statistical difference between PRG and controls, POG and PRG (P<0.05). | | 16 D |
| Chen et al.  (2020) [62] | LI4, PC6, SI3, TE6 | 1. TEA: before, during, and after surgery.  2. sham-TEAS: before and after surgery. | 30 min before anesthetic induction, throughout the surgery and at 6, 24, and 48 hours after the surgery | The VAS scores one month after surgery were significantly lower in the TEAS group than those in the sham-TEAS group (P < 0.001). | | 30 D |
| Yu  (2020) [48] | lung area | 1/D, 4W | The whole course of treatment | The score of KPS was significantly higher (P<0.05). | | NA |
| Sun et al.  (2020) [61] | SP10, SP6, ST36 | 2/D, 5D | After surgery, when the patient was awake, and all vital signs were stable | The total score of FACT-L and KPS were higher (P<0.05). | | NA |
| Zhang et al.  (2020) [45] | C014, AT14, TF4, AH6a, Ashi-points; BL13, EX-B1, BL43, RN22 | 1. Auricular acupoint: press 4-5/2D, 4W  2. Point application: 2/D, 4W | The whole course of treatment | The scores of QOL and KPS were higher(P<0.01). The scores of SAS and SDS were lower(P<0.01). | | NA |
| Wang  (2020) [41] | LI4, LR3; AT4, TF4, AH6a | 2/W,8W | The whole course of treatment | The scores of QL, PF, RF, EF, CF and SF in QLQ-C30 were higher, and the scores of FA, NV, DY, SL, AP, CO, DI and FI were lower (P<0.05). | | NA |
| Liu et al.  (2020) [42] | C012, TF4, AH6a, AT4, AT3, Ashi-points | 2/W, 8W | The whole course of treatment | The decrease of the NRS score and the increase of the KPS score were more significant (P<0.05). | | NA |
| Ma et al.  (2019) [50] | BL13, EX-B1 | 1/2D, 10D | The whole course of treatment | The KPS score and LCQ score were improved remarkably (P<0.05). | | NA |
| Zhou et al.  (2019) [51] | RN12, RN10, BL12, ST21 | 2/D, 1W | 2 H before chemotherapy to 2 D after chemotherapy | The acute vomiting degree of patients was significantly lower (P<0.05). | | NA |
| Guo et al.  (2019) [43] | C014, C015, C010, TF4, AT4, AH6a, HT7, ST36, KI1 | 1/D, 2W | The whole course of treatment | After treatment, there were statistically significant differences in PSQI scores of the 4 groups (P<0.05), suggesting that the effect of auricular acupoint + moxibustion treatment group was the most obvious. | | NA |
| Wang et al.  (2019) [53] | ST36, RN12, PC6 | 1/D, 3W | ①30 min before chemotherapy  ②30 min after chemotherapy | The KPS scores were obviously lower (P<0.05). | | NA |
| Yin  (2019) [49] | KI1, ST36, PC6 | 1/D, 1W | The whole course of treatment | The changes in the KPS score were better than those in the control group (P<0.05). | | NA |
| Deng et al.  (2019) [64] | ST36 | 1/D, 2W, discontinues for 1 W, 6 W | The whole course of treatment | The index of stability of KPS of the treatment group was obviously higher(P<0.05). | | NA |
| Hou et al.  (2017) [63] | RN6, BL17, ST36 | on the 1^st^, 2^nd^, 3^rd^, 5^th^, 8^th^, 11^th^, 14^th^, and 28^th^ days | The whole course of treatment | All the dimension indicated statistically significant differences(P<0.05), with the exception of sensory fatigue (P=0.50). However, TEA Group (Mean =2.48) had a lower sensory fatigue score. | | NA |
| Cheng et al.  (2017) [54] | LI4, RN6, ST36, KI3, SP6 | 8 sessions that were 45 min in duration and administered over 4 W | The whole course of treatment | The FACT-L score was higher (P<0.05). The BFI-C score was lower (P<0.05). | | 14 D |
| Lu et al.  (2017) [44] | C015, TF4, AT3, AT4, L04 | 4-5 /D, 4W | First day after radiotherapy | There was no significant difference in the average AIS score between the two groups (P>0.05). | | NA |
| Fan et al.  (2017) [56] | PC6, LI4, ST36, GB34, SP6 | 1/D, 2W | The whole course of treatment | The NRS score was lower (P<0.05). | | NA |
| Wang et al.  (2017) [57] | DU14, RN12, RN4, ST36, LI4, SP10, SP6 | 1/D, until out of hospital | 12 hours after the operation | The QLQ-C30 scores were better (P<0.05). | | NA |
| Pei et al.  (2017) [65] | BL17, BL19 | 1/D, 1W | The whole course of treatment | The KPS and FACT-L score were higher (P<0.05). | | NA |
| Ou et al.  (2016) [46] | BL12, BL13, BL15, PC2, BL43, LU1, LU5, tender point; C014, C015, C07, TG2p, C018, TG4, TG3, AH10; BL23, ST36, RN4, DU4 | 1/D, 10-15D | The whole course of treatment | The improvement of the KPS score was better (P <0.05). | | 30 D |
| Wei et al.  (2016) [70] | BL13, BL23, RN17, ST36, Ashi-points | 1/W | The whole course of treatment | The remission rate of cancer pain was higher than that of the control group (P<0.05). | | NA |
| Wu et al.  (2016) [72] | RN8, KI1, RN6, BL13 | 1/D, 3W | The whole course of treatment | The total score of RPFS and the scores of all dimensions were significantly lower (P<0.05). | | NA |
| Liu  (2016) [67] | PC6, ST36, RN12 | 6/D, 3D | The whole course of treatment | The proportion of mild fatigue and below was significantly lower (P<0.05)**.** | | NA |
| Shen et al.  (2016) [59] | KI6, BL62, HT7, EX-HN3, EX-HN1 | 1/D, 4W | The whole course of treatment | The score of NRS, SAS, PSQI and SDS of the patients in observation group were significantly lower (P<0.05 or P<0.01) | | NA |
| Li et al.  (2014) [66] | BL17, BL19 | 1/D, 1W | The whole course of treatment | The KPS score was higher (P<0.05). | | NA |
| Randolph et al. (2016) [60] | LI4, GB34, TE8, GB36 | 2/D, 1W | The first 7 postoperative days | There was a trend for lower VAS score between postoperative days 2 and 6 (P>0.05). | | NA |

Abbreviations: TEAS, transcutaneous electrical acupoint stimulation; EA, Electroacupuncture; SA, sham acupuncture; VATS, video assisted thoracic surgery; PRG, pre-chemotherapy acupuncture group; POG, post-chemotherapy acupuncture group; KPS, Karnofsky Performance Status; QLQ-C30, European Organization for Research and Treatment of Cancer Quality of Life Questionnaire; FACT-LCS, the Functional Assessment of Cancer Therapy Lung Cancer Subscale; SF-36, the MOS item short from health survey; LCQ, Leicester Cough Questionnaire; FACT-L, Functional Assessment of Cancer Therapy-Lung; NRS, Numerical Rating Scale; VAS, Visual Analogue Scale; BPI-C, Brief Pain Inventory-Chinese Version; MAT, MASCC (Multinational Association of Supportive Care in Cancer) Antiemesis Tool; INVR, Index of Nausea and Vomiting and Retching; PSQI, Pittsburgh Sleep Quality Index; AIS, Athens Insomnia Scale; SAS, Self-Rating Anxiety Scale; SDS, Self-Rating Depression Scale; BFI-C, Brief Fatigue Inventory-Chinese Version; PFS-R, The Revised Piper Fatigue Scale.
