# Supplementary material for: Acupuncture for adult lung cancer of patient-reported outcomes: A systematic review and meta-analysis
Source: Front Oncol. 2022 Sep 2;12:921151. doi: 10.3389/fonc.2022.921151 (PMC9479629; doi:10.3389/fonc.2022.921151)
Supplement: Supplementary file 6 [file Table_6.docx]

| **Supplementary Table 6 \|** The results of meta regression in patients of postoperative pain measured by the combination of NRS and VAS with different variables. | | | | |
| --- | --- | --- | --- | --- |
| **Outcome** | **PRO** | **Study** | **Variables** | **P>\|t\|** |
| Pain  (postoperative pain) | NRS+VAS | Liu 2021  Chen 2020  Randolph 2006 | Publication year | 0.606 |
|  |  |  | Country of publication | / |
|  |  |  | Duration time | 0.767 |
|  |  |  | TNM stage | 0.767 |
|  |  |  | Acupuncture technique | 0.238 |
|  |  |  | Couse of treatment | 0.238 |
|  |  |  | Frequency of treatment | 0.238 |

Abbreviations: NRS, Numerical Rating Scale; VAS, Visual Analogue Scale; TNM, tumor-node-metastasis.
