# Supplementary material for: Acupuncture for adult lung cancer of patient-reported outcomes: A systematic review and meta-analysis
Source: Front Oncol. 2022 Sep 2;12:921151. doi: 10.3389/fonc.2022.921151 (PMC9479629; doi:10.3389/fonc.2022.921151)
Supplement: Supplementary file 7 [file Table_7.docx]

| **Supplementary Table 7 \|** The results of meta regression in patients of sleep disturbances measured by PSQI with different variables. | | | | |
| --- | --- | --- | --- | --- |
| **Outcome** | **PRO** | **Study** | **Variables** | **P>\|t\|** |
| Sleep disturbances | PSQI | \| Shen 2016 \| \| --- \| \| Kou 2021 \| \| Bai 2021 \| \| Yang 2021 \| | Publication year | 0.457 |
|  |  |  | Country of publication | / |
|  |  |  | Duration time | 0.017* |
|  |  |  | TNM stage | 0.488 |
|  |  |  | Acupuncture technique | 0.274 |
|  |  |  | Couse of treatment | 0.274 |
|  |  |  | Frequency of treatment | 0.065 |

*P<0.05.

Abbreviations: PSQI, Pittsburgh Sleep Quality Index; TNM, tumor-node-metastasis.
