# Supplementary material for: Acupuncture for adult lung cancer of patient-reported outcomes: A systematic review and meta-analysis
Source: Front Oncol. 2022 Sep 2;12:921151. doi: 10.3389/fonc.2022.921151 (PMC9479629; doi:10.3389/fonc.2022.921151)
Supplement: Supplementary file 8 [file Table_8.docx]

| **Supplementary Table 8 \|** The results of meta regression in patients of fatigue measured by PFS-R with different variables. | | | | |
| --- | --- | --- | --- | --- |
| **Outcome** | **PRO** | **Study** | **Variables** | **P>\|t\|** |
| Fatigue | PFS-R | \| HOU 2017 \| \| --- \| \| Liu 2020 \| \| Wu 2016 \| \| Yang 2021 \| | Publication year | 0.125 |
|  |  |  | Country of publication | / |
|  |  |  | Duration time | 0.130 |
|  |  |  | TNM stage | 0.556 |
|  |  |  | Acupuncture technique | 0.130 |
|  |  |  | Couse of treatment | 0.182 |
|  |  |  | Frequency of treatment | 0.326 |

Abbreviations: PFS-R, The Revised Piper Fatigue Scale; TNM, tumor-node-metastasis.
